# Live the Experience: Mental Health Simulation Training for New Starters to Psychiatry in East London NHS Foundation Trust (ELFT)

**DOI:** 10.1192/bjo.2024.319

**Published:** 2024-08-01

**Authors:** Alisha Patel, Haitham Ismail, Lorena Valdearenas

**Affiliations:** East London NHS Foundation Trust, London, United Kingdom

## Abstract

**Aims:**

This project aimed to create and deliver a simulation-based course to improve trainees’ knowledge, practical skills and confidence as well as leadership and multidisciplinary-team working. We evaluated the effectiveness of this training and simulation as a learning experience. Simulation in psychiatry is a relatively new field compared with other specialities. Literature shows that experiential learning in psychiatry is effective for developing clinical and communication skills for doctors, and confidence in leadership. It is vital we work towards the National Health Service Long Term Plan for improving mental health care for those with serious mental illness which includes better training for doctors. This course was designed to enhance the ELFT training programme focusing on applications of theoretical knowledge.

**Methods:**

A simulation-based course was delivered to core trainees and general practitioner trainees at induction to psychiatry. This was based on the Royal College of Psychiatrists curriculum and input from our People Participation team to ensure authenticity of scenarios. We surveyed trainees to inform the development of our pilot in February 2022 and subsequently developed two half-day courses facilitated in August 2022 following feedback. The scenarios we created were: risk assessment, section 5(2) Mental Health Act (MHA) assessment, managing agitation and violence, escalating concerns to a senior, section 136 MHA assessment, seclusion review, discussion with medical registrar for physical health concerns, collateral history and information-giving in child psychiatry. We used a structured debrief model (what went well, what could you have done differently, what was the ‘golden moment'?) and provided relevant teaching. Service users joined the debrief to share their perspectives and lived experiences. We collected and analysed quantitative and qualitative feedback.

**Results:**

Ten trainees attended the pilot course, followed by eleven on day 1 and nine on day 2 in August 2022. Results from questionnaires revealed post-course, 100% of participants felt more confident in their psychiatric skills and found this experience to be valuable for clinical practice. 100% would recommend this simulation to others. Qualitative data showed participants thought scenarios were realistic, the environment was supportive and feedback was comprehensive. They also appreciated the service user involvement.

**Conclusion:**

Trainees reported simulation provided a safe and engaging environment to learn practical skills which better prepared them for work. This course is now embedded into the ELFT induction programme and enables doctors to develop their confidence and have a better understanding of service user perspectives. Future development of this course will involve allied health professionals.

